# The Evolution of the Intrinsic Flexural Strength of Jute Strands after a Progressive Delignification Process and Their Contribution to the Flexural Strength of PLA-Based Biocomposites

**DOI:** 10.3390/polym16010037

**Published:** 2023-12-21

**Authors:** Francisco J. Alonso-Montemayor, Francesc X. Espinach, Quim Tarrés, Manel Alcalà, Marc Delgado-Aguilar, Pere Mutjé

**Affiliations:** LEPAMAP-PRODIS Research Group, University of Girona, C/Maria Aurèlia Capmany 61, 17003 Girona, Spain; francisco.espinach@udg.edu (F.X.E.); joaquimagusti.tarres@udg.edu (Q.T.); manuel.alcala@udg.edu (M.A.); m.delgado@udg.edu (M.D.-A.)

**Keywords:** jute strands, delignification polylactic acid, biocomposites, flexural strength behavior, intrinsic flexural strength

## Abstract

Biocomposites from poly-(lactic acid) (PLA) and jute strands were prepared, and their flexural strength was analyzed. Jute strands were submitted to a progressive delignification process and the resulting morphology, composition, and crystallinity index were evaluated. Then, PLA biocomposites comprising 30 wt% of jute strands were produced and characterized under flexural conditions. The delignification processes decreased the lignin content and progressively increased the cellulose content. All this resulted in an enhancement of the composite flexural strength. A modified rule of mixtures, and the relation between tensile and flexural properties were used to determine the intrinsic flexural strength (of the jute strands) and their correlation with their physic-chemical characteristics. Equations correlating the intrinsic flexural strength with the crystallinity index, the cellulose content, and the microfibril angle were proposed. These equations show the impact of these properties over the intrinsic properties of the fibers and can help researchers to select appropriate fibers to obtain accurate properties for the composites. Jute strands show their value as reinforcement by increasing the flexural strength of the matrix by 70% and being less expensive and more environmentally friendly than mineral reinforcements. Together with the profitability and the environmental advantages, the mechanical results suggest that these PLA biocomposites are suitable for specific products of different market sectors.

## 1. Introduction

The growing environmental awareness of society is shifting industries towards higher utilization of bio-based and biodegradable raw materials to manufacture their products [1]. In this context, natural fibers are playing an important role for composite manufacturers, either to be used as a substitute for mineral fibers or to lessen the final product’s cost. Anyhow, the decision will be subjected to the composite or biocomposite performance. It is known that the mechanical properties of composites mainly function as a result of the intrinsic properties of their components, the fiber-matrix interface, the fiber content, and the surface morphological characteristics of the fibers [2]. At a critical fiber length, the stress is transferred from the matrix to the fibers, resulting in a strengthening of the matrix [3]. In principle, plant fiber-reinforced composites should have similar applications as short glass fiber (GF)-reinforced composites (e.g., structural, automotive, or furniture sectors) [4].

PLA is one of the polymeric matrices that has called researcher interest in the last few years. PLA is a transparent, highly crystalline, and industrially compostable polymer. This polymer can be obtained from petroleum sources or bioresources. Thus, the production of biobased PLA involves fewer fossil resources as compared to polymers exclusively obtained from petroleum sources [5]. In order to ensure a robust environmental impact of the matrix, the researchers must be aware of its origin and the chemical process used to obtain it [1,5]. In addition, PLA exhibits high mechanical strength, thermoplastic behavior, biocompatibility, and good processability [6]. PLA has several industrial applications such as in packaging, textiles, biomedicine, and structures [1]. 

Cellulose fibers can be classified as non-wood fibers and wood fibers [7]. From an origin point of view, the lignocellulosic fibers can be obtained from strands of annual plants (e.g., abaca, flax, hemp, jute, sisal, and cotton), wood fibers (e.g., softwood and hardwood), fibers obtained from agricultural residues (e.g., bagasse, corn, and colza), forest residues (e.g., young wood obtained mainly from pruning fruit trees), and secondary fibers from recycled paper and board. These lignocellulosic fibers are characterized by a specific chemical composition (cellulose, hemicellulose, lignin, and extractives) with a determined length-diameter (l/d) ratio. In turn, the lignocellulosic fibers can be single strands or bundles of strands, depending on the ability of the extraction method to remove the glue components (e.g., pectin and lignin) binding the single strands. Differentiating single strands from the bundles is important since the strands exhibit higher intrinsic strength and stiffness than the bundles. Subsequent exposition of the strands to sodium hypochlorite solutions led to significant variations in their chemical composition since practically all lignin and a considerable fraction of hemicelluloses can be removed after a critical delignification stage using a non-selective solvent such as sodium hypochlorite [8], instead of sodium chlorite, which preserves the hemicelluloses. The removal of the soft and amorphous phases from the fibers not only strengthens these, but also allows the strong and highly crystalline cellulose microfibrils to interact with the matrix, enhancing the composite mechanical performance [1].

Among the strands, jute is one of the most promising and highly commercially available fibers to reinforce PLA. Jute strand-reinforced polymer composites have already been used to manufacture automobile interior decoration and architectural furnishing [9]. Jute belongs to the *Malvaceae* family, *Corchorus* spp., which comprises around 100 species, and is currently one of the cellulose fibers with the highest production rate and has an inherently low strain at break of about 1.7% that may provide high mechanical strength to the reinforced composites [6]. Together with hemp, jute has better physical and mechanical properties when compared to other natural fibers. Besides, using cheap reinforcement can deliver economic competitiveness to the composite [10].

**Figure 1:**
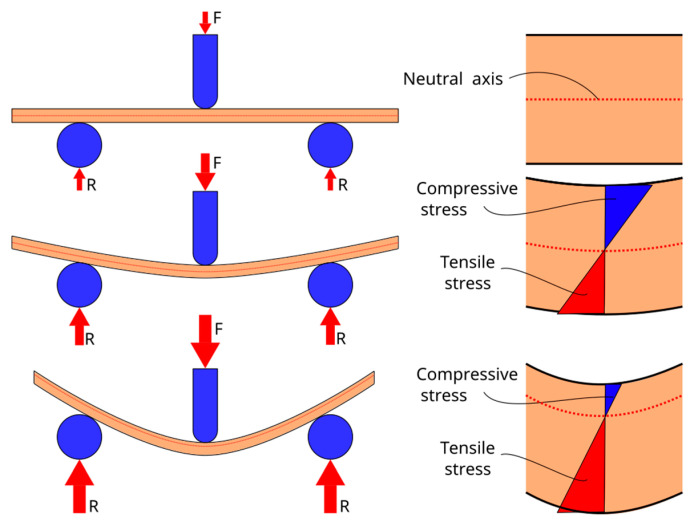
Evolution of the position of the neutral axis against specimen strain, showing the areas of the section of the specimen under compressive and tensile stresses. (R refers to the reaction at the supports and F refers to the applied loads”.

Short fiber-reinforced composites show anisotropic behavior. Usually, the tensile properties are lower than the flexural properties. As Figure 1 depicts, during a flexural test the composite is subjected to compressive and tensile forces above and below the neutral axis, respectively [11,12]. Depending on the application, cellulose fiber-reinforced composites must be designed to offer the best response to loads following a preferable direction. This is of great importance when a novel material is introduced in the market since it needs to meet the industrial requirements of specific applications. Specifically, flexural properties are significantly important and relevant for engineers when predicting the potential of the material to be applied in structural, semi-structural, construction, and other similar commercial areas. This is explained by the fact that flexural or bending conditions are far more common than situations with tensile loads only, making designers especially interested in predicting the behavior of the materials at flexural loads [13].

By the analysis of the composite flexural strength ($\sigma_{f}^{C}$) it is possible to model the mechanical behavior to obtain the intrinsic fiber flexural strength ($\sigma_{f}^{F}$) and the flexural coupling factor ($f_{C}$) as main outcomes. The modified rule of mixtures is depicted according to Equation (1) [14]:

(1) $$\sigma_{f}^{C}=f_{C}\sigma_{f}^{F}V^{F}+\left(1-V^{F}\right)\sigma_{f}^{m*}$$

where, $\sigma_{f}^{C}$ is the composite flexural strength, $\sigma_{f}^{F}$ the intrinsic fiber flexural strength, $\sigma_{f}^{m*}$ is the contribution of the matrix to the $\sigma_{f}^{C}$ at the composite break, $V^{F}$ the fiber volume fraction, and $f_{C}$ the flexural coupling factor. From this equation, the flexural strength of a composite can be predicted using its dependency on the matrix characteristics, fiber content, fiber distribution, and morphology of the fiber. It is also worth mentioning that the intrinsic properties of the strands will depend on their origin, soil characteristics, and climatic conditions; these conditions will affect their chemical composition and morphology, including parameters such as cellulose content (Cel%), crystallinity index (CI), degree of polymerization, density ($\rho^{F}$), diameter (D), and microfibrillar angle (MFA) of cellulose at the cell wall of the strands [14].

In this work, jute strands were subjected to different delignification stages using a sodium hypochlorite solution. The strands were chemically characterized, and their crystallinity index was determined. Later, PLA composites of PLA comprising 30 wt% of these jute strands were prepared and tested under flexural conditions. A modified rule of mixtures (Equation (1)) was used to obtain the intrinsic flexural strength of the jute strands. The correlation between the intrinsic flexural strength of the strands and their physic-chemical characteristics (CI, Cel%, and MFA) is explored and different equations are proposed. To the best of our knowledge, this correlation has been seldom reported in the literature and never for jute strands.

## 2. Materials and Methods

### 2.1. Materials

The composites were prepared utilizing polylactic acid (PLA) (Ingeo Biopolymer 3251D), with a melt flow index (MFI) of 35 g/10 min at 190 °C and a load of 2.16 kg, which was kindly provided by Natureworks LLC (Blair, NE, USA). Jute strands were kindly supplied by CELESA S.A. (Tarragona, Spain). The raw jute strands have a diameter and mean weighted length of 22.9 μm and 353.0 μm, respectively [15]. All the chemical reagents used for strand characterization, extraction, and bleaching were supplied by Scharlau, S.L. (Sentmenat, Spain).

### 2.2. Delignification Treatments

**Figure 2:**
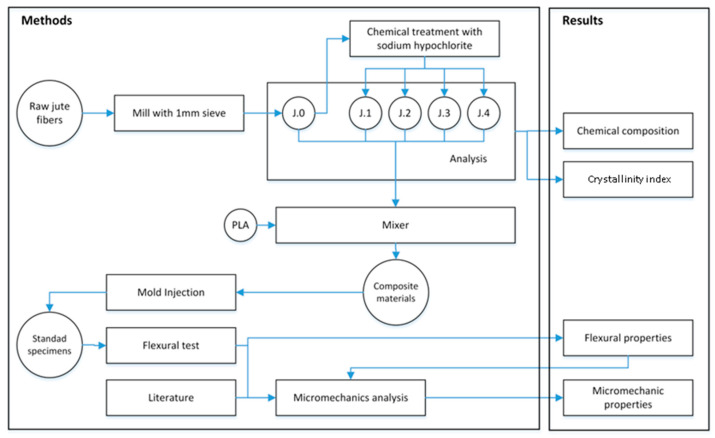
Flowchart of the experimental procedure.

The raw jute strands (J.0) were milled using a knife mill and passed through a 1 mm mesh. The jute strands were subsequently delignified with a solution with 8% sodium hypochlorite at 70 °C for 30 min, per stage. This is to say, that raw jute strands (named J.0) were subjected to four subsequent delignification stages (named J.1 to J.4, respectively). The number following “J” indicates the number of delignification stages. After each delignification stage, the strands were washed with distilled water to remove the residual sodium hypochlorite and dried at 105 °C. Figure 2 shows the flowchart of the experimental procedure. The mixing process, the preparation of the biocomposites, and the characterization procedures are addressed in further sections. 

### 2.3. Kappa Number and Chemical Composition

The Kappa number (KN) of the jute strands was determined following the ISO 302:2004 to evaluate the lignin presence. The TAPPI standards T204 cm-97, T413 om-93, and T-222 [16,17,18] were used to determine the content of lignin, extractives, and ashes. The amount of holocellulose was estimated from the difference between the total weight and the sum of lignin, extractives, and ashes. The Cel% was evaluated according to the TAPPI-T-212-om-12 standard [19].

### 2.4. The Preparation of the Fully Biodegradable Composites

The jute strands were homogeneously incorporated at 30 wt% to the PLA matrix using a Gelimat multi-kinetic mixer. Both strands and matrix were incorporated into the mixing chamber at low speed (300 rpm), which was gradually increased up to 2500 rpm. At this stage, the polymer matrix was molten and blended with jute strands. The process lasted from 3 to 4 min. Each material was discharged and milled using a knife mill. Before injection molding, the pellets were dried in an oven to remove moisture. Standard specimens for tensile testing were obtained in an Allrounder-220M injection machine fabricated by ARBURG (Eschweiler, Germany) according to UNE-EN ISO 178:2001 standard [20]. Before flexural testing, specimens were placed in a climatic chamber at 23 °C and 50% relative humidity for 48 h.

### 2.5. Flexural Characterization

The characterization of the bending properties of the PLA composite specimens was carried out following the UNE-EN ISO 178:2001 standard dimensions [21]. Ten samples of each PLA composite, reinforced with J.0, J.1, J.2, J.3, and J.4 were tested using an Instron 1122 universal testing machine equipped with a 5 kN load cell. A load element with a radius of 5 mm and supports located at 50 mm between them was used. ANOVA analyses of the results were made with R^®^ and RCommander at a 95% confidence interval.

### 2.6. Densities Determination

According to ISO 1183-1 [21], a pycnometer was used to obtain the empirical values needed to determine the density of the composites ($\rho^{C}$). Distilled water was used as a reference liquid. The composite density was calculated by Equation (2) [22].

(2) $$\rho^{C}=\frac{w^{C}}{V-w^{H_{2}O}\rho^{H_{2}O}}$$

where V is the total volume of the pycnometer and $\rho^{H_{2}O}$ is the density of the water. The weight of the composite and water were represented by $w^{C}$ and $w^{H_{2}O}$, respectively. The $\rho^{F}$ identity described by Equation (3) was deduced from the Equation (2) [22].

(3) $$\rho^{F}=\frac{w^{F}\rho^{C}\rho^{M}}{w^{C}\rho^{M}-w^{M}\rho^{C}}$$

where, $\rho^{M}$ is the density of the matrix. The weight of the fiber and the matrix were signed as $w^{F}$ and $w^{M}$, respectively. The $\rho^{F}$ calculation was used to estimate the $V^{F}$, which is a parameter that can be calculated from Equation (4) [22].

(4) $$V^{F}={\left(1+\frac{\rho^{F}\left(1-Fiber\;content(wt\%)\right)}{\rho^{M}x^{F}}\right)}^{-1}$$

### 2.7. Fiber Recovering from Composites

The obtained biocomposites were submitted to Soxhlet extraction in the presence of dichloromethane, dissolving PLA with the purpose of recovering the fibers inside the matrix. This extraction was performed with grinded biocomposites for 24 h. Finally, the fibers were washed with distilled water and stored at 4 °C for morphological analysis [14].

### 2.8. The Morphological Analysis of Fibers

Morphological analysis was carried out using MorFi analyzer (Techpap SAS, Grenoble, France), equipped with CCD video camera. About 30,000 fibers were analyzed by the software MorFi v9.2. Among other parameters, this software was able to calculate the mean fiber length, the mean diameter and the fines percentage (fibers shorter than 76 μm). All characterizations were performed in triplicate [14].

### 2.9. The Evaluation of the Intrinsic Flexural Strength of the Reinforcements and Their Contribution to the Flexural Strength of the Composites

In addition, the well-known limitations associated with the measurement of the intrinsic properties of strand-reinforced composites would imply [22] that the use of mathematical models to estimate such intrinsic properties was chosen since the fiber deformation inside a composite is considerably lower than the minimum strain considered by any empirical approach (e.g., the single fiber tensile test has a minimal strain of 0.6 mm, while the fiber strain inside the polymer matrix is around of 0.35 mm). The literature presents a wide variety of models that can be used for that purpose. Nonetheless, one of the more elegant, due mainly to its simplicity, is a modified rule of mixtures. Nevertheless, the $\sigma_{f}^{F}$ and $f^{C}$ are unknown values, leaving Equation (5) unsolved. However, a fiber flexural strength factor (FFSF) can be estimated throughout Equation (5) to know the neat contribution of the fibers to the $\sigma_{f}^{C}$. As can be seen, the FFSF can be calculated from known parameters [23].

(5) $$FFSF=\frac{\sigma_{f}^{C}-\left(1-V^{F}\right)\sigma_{f}^{m*}}{V^{F}}=f_{C}\sigma_{f}^{F}$$

Anyway, the $\sigma_{f}^{F}$ of the fibers remains unknown and it cannot be experimentally obtained for non-wood fibers and single strands. The first way used to estimate the $\sigma_{f}^{F}$ is $\sigma_{f}^{F}=\sigma_{t}^{F}\frac{FFSF}{FTSF}$ which is based on the ratio of FFSF and the fiber tensile strength factor (FTSF), where $\sigma_{t}^{F}$ is the fiber tensile strength [24]. These factors represent the contribution of the fibers to the respective composite strength values. Meanwhile, the second way $\sigma_{f}^{F•}=\sigma_{t}^{F}\frac{\sigma_{f}^{C}}{\sigma_{t}^{C}}$, is based on the ratio of the $\sigma_{f}^{C}$ and the composite tensile strength ($\sigma_{t}^{C}$) [25]. Finally, once $\sigma_{f}^{F}$ or $\sigma_{f}^{F•}$ are known, the respective flexural coupling factors ($f_{C}$ and $f_{C}^{•}$) can be calculated from Equation (1). It is known that the morphology of the fibers, mainly their length distribution and diameter, impacts the mechanical properties of a composite. In this case, the authors link the tensile and flexural properties of the composites and the fibers to obtain the intrinsic flexural strength of the fibers. The intrinsic tensile strength of such fibers was obtained by solving Kelly and Tyson’s equation that takes into account the mean diameter and the length distribution of the reinforcements. Then, while such morphologic parameters are not implicitly used in the model used to obtain the intrinsic flexural strength of the reinforcements, they are explicitly used because the value of the intrinsic tensile strength was obtained by taking into account such morphologic properties [25].

## 3. Results and Discussion

Understanding the microstructure and chemical composition of the cellulose fibers is needed to design and develop reinforced polymer composites. The delignification of virgin jute generates four types of strands that have been evaluated in terms of their bending resistance. As Table 1 shows, the progressive delignification not only produced changes in the surface of the strand, affecting the strength of its interface with PLA but also in the chemical composition of the strands. It is important to highlight that the lignin content follows a lineal correlation (0.996 R^2^) with the KN described by 0.4743(KN)+0.0591, which is useful for predictions.

Broadly speaking, the cellulose content increases gradually, while a fraction of the hemicelluloses is carried along with the lignin. Therefore, each value of composite flexural strength must be considered relative to a specific composite. That is to say, the generated strands have a different chemical composition and $\rho^{F}$ values, working with the same reinforcement weight load, that increase as the lignin and hemicelluloses contents decrease, and while the cellulose content and the CI increase, as indicated in Table 1. The evolution of the D of the strands indicates that these were slightly compacted. This compaction can be associated with the structural rearrangement implied by the chemical changes caused by the delignification stages.

### 3.1. The Flexural Properties of PLA Matrix and Jute Strand-Reinforced Composites

Table 2 shows the flexural properties of PLA and its composites, reinforced with 30 wt% of jute strands and delignified strands, which were assessed in terms of $\sigma_{f}^{C}$ and the matrix flexural strength ($\sigma_{f}^{M}$), elongation at break ($\varepsilon_{f}^{M}$) and the composite ($\varepsilon_{f}^{C}$) under flexural load, and the $\sigma_{f}^{M*}$. Additionally, for further analysis, the $V^{F}$ of the reinforcing jute strands was calculated for each delignification stage.

The ANOVA analysis at a 95% confidence rate shows that the first stage of delignification had a statistically significant impact on the flexural strength of the composites. A second delignification treatment show no significant impact on the same property. A third delignification treatment returned flexural strength for the composites with significant differences to the other composites. A fourth delignification treatment, showed a similar impact to that of a one or two stage treatment. All of the composites show significative differences in the flexural strength of the matrix. In the case of tensile strength, the delignification treatment showed an impact on the tensile strength of the composites up to three stages. The results obtained with the fourth stage treatment is statistically similar to a two or three stage treatment. All the delignification stages showed a similar impact on the strain at the break of the composites.

**Figure 3:**
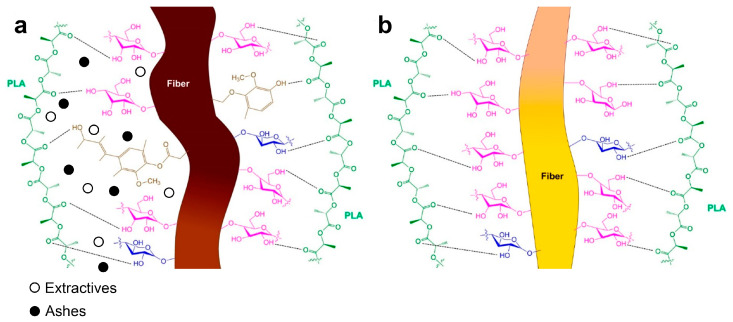
Schematic of possible chemical and electrostatic interactions between the PLA matrix and a raw jute strand with 14% lignin (**a**) and a fully delignified jute strand (**b**).

The enhancement of the $\sigma_{f}^{C}$ of the PLA composites with the incorporation of delignified jute strands can be attributed to a stronger interphase between the matrix and the jute strands achieved by the removal of lignin and hemicelluloses after the exposition to the sodium hypochlorite solution [28]. The fiber-matrix interface is produced by the capacity of the PLA functional groups to interact with those of the jute strands fusing hydrogen bonds and van der Waals interactions, as Figure 3 shows [29]. It has been explained that the higher aspect ratio and cellulose content in the delignified strands provide enhanced composite flexural properties since increased active surface and more cellulose content mean higher hydroxyl groups available at the fiber surface for bonding with the matrix [3].

Before any delignification process, lignin, extractives, and holocellulose are usually found on the most superficial layer of the strands [30]. According to the Börås–Gantenholm model, which represents the chemical distribution on the surface of a typical chemi-thermo mechanical pulp fiber, the largest area corresponds to lignin (28%) and extractives (32%), while only 40% of the remaining surface was covered with holocellulose [31]. For fibers with a high number of extractives, the waxy substances and pectin should cap the functional groups of raw cellulose fibers [1,2]. From this, it can be deduced that the raw jute strands did not present enough extractives that hinder the interactions between their functional groups and those of the PLA since the flexural strength was enhanced with the incorporation of the jute strands prior to being subjected to sodium hypochlorite treatment.

The enhancement of the $\sigma_{f}^{C}$ of the PLA composites reinforced with jute strands is in line with the reported $\sigma_{t}^{C}$ trend [15], presenting a good coefficient of correlation of 0.989 R^2^ and higher strength values. Similar enhancement trends have been reported for the $\sigma_{f}^{C}$ of PLA composites reinforced with 30 wt% of raw and alkali-treated hemp fibers [32]. 

For subsequent delignification stages, the incorporation led to a stabilization or “plateau” of the $\sigma_{f}^{C}$ around 100 MPa. Considering that sodium hypochlorite solutions remove surface lignin and hemicellulose from the cellulose fibers [1], it can be speculated that subsequently exposing the fibers to the sodium hypochlorite solution may remove a fraction of cellulose, reducing the fiber capacity to reinforce and leading to a plateau effect on the $\sigma_{f}^{C}$. However, the number of ruptures, voids, and dislocations within the reinforcing jute strands, and the changes in the strand’s polymerization degree after being exposed to alkali treatment (from 2500 for virgin to 1020 for alkali-treated) [14,33,34,35] are factors that also determine flexural strength and also need to be considered. Anyway, it seems to be that the subsequent delignification of the jute strands is not required to enhance the $\sigma_{f}^{C}$.

On the other hand, it is important to highlight that the jute strand-reinforced PLA composites achieved around 70% of the $\sigma_{f}^{C}$ values of a commercially available and another reported PLA composite reinforced with 30 wt% GF, whose values of $\sigma_{f}^{M}$ were 82 MPa [26] and 62 MPa [27], respectively, and considerably higher than PP composites reinforced with 30 wt% of chopped GF ($\sigma_{f}^{C}=79.70\pm 0.80$ MPa, respectively) [36]. These results indicate that the delignified jute strand-reinforced PLA composites could compete with GF-reinforced PP composites and be a complementary option to GF-reinforced PLA composites, especially when other advantageous properties of the jute strand reinforcement are required (e.g., low density and high specific strength).

### 3.2. The Evaluation and Analysis of the Intrinsic Flexural Properties

**Figure 4:**
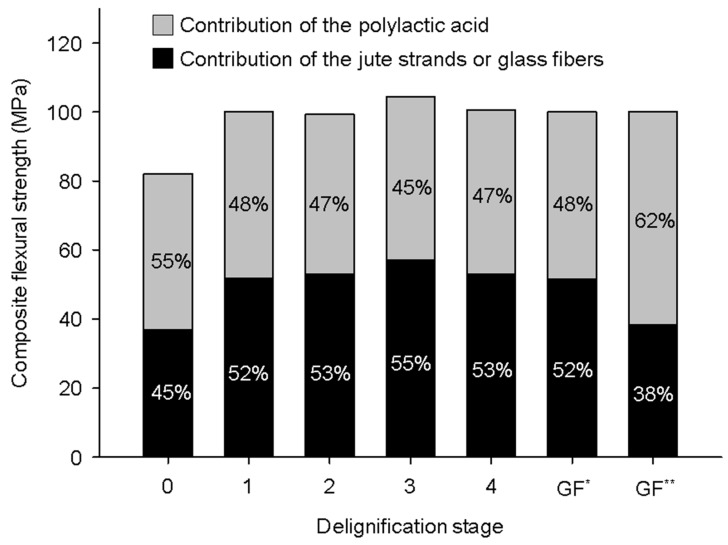
Percentage contributions of the PLA and delignified jute strands or GF to the $\sigma_{f}^{C}$. (GF^*^ and GF^**^ are values, respectively, obtained from the data reported by [26,27]).

Figure 4 shows the contribution of the PLA and jute strands to the $\sigma_{f}^{C}$. It was found that the delignified jute strands contributed more than 50% of the $\sigma_{f}^{C}$, while the contribution of the virgin jute strands was lesser. Moreover, it was found that the jute strands exhibited strength contributions comparable to the abovementioned reported GF-reinforced composites. This difference in contribution depends not only on the type of strand and $\sigma_{f}^{F}$ magnitude, but also on the reinforcing capability of the strands, which is expressed by the $f_{C}$. However, in the case of GF-reinforced PLA composites, it is necessary to consider that PLA could suffer hydrolysis during the processing of the composites [27] which could affect the estimation of $\sigma_{f}^{F}$ and $f_{C}$.

After corroborating that the $\sigma_{f}^{C}$ and $\sigma_{t}^{C}$ present a good correlation, there are at least two methods to estimate $\sigma_{f}^{F}$ values apart from a modified rule of mixtures approach. These methods assume that similar $f_{C}$ values can be obtained from the tensile and flexural mechanical properties. Table 3 compiles the $\sigma_{f}^{F}$ and $\sigma_{f}^{F•}$ obtained from Equations (6) and (7), respectively.

It was found that $\sigma_{f}^{F}$ and $f_{C}$, respectively, exhibit high correlations and similar percentages above 95% with $\sigma_{f}^{F•}$ (0.992 R^2^) and $f_{C}^{•}$ (0.988 R^2^). These $f_{C}$ values were considered high for cellulose fiber-reinforced polymers, implying that the fiber-matrix interface was strong in general. Meanwhile, for the GF-reinforced PLA composites, the Equation (7) approach cannot be replaced by Equation (6) since the $f_{C}$ was too different from $f_{C}^{•}$, indicating that there could not be a good correlation between the tensile and flexural behavior. These results indicate that Equation (7), recently proposed by our research group, led to a more similar $\sigma_{f}^{F}$ estimation than Equation (6), which has been proved with other cellulose fiber-reinforced composites [37], when applied to delignified jute strands-reinforced PLA composites. Moreover, the results show that the delignification stages not only provoked chemical and morphological changes in the strands but also strengthened them. In the LEPAMAP-PRODIS research group it was considered that fiber very well, well, normally, poorly, and badly bonded to the polymer matrix corresponding to the $f_{C}$ value ranges of 0.19–0.21, 0.17–0.18, 0.15–0.16, 0.13–0.14, and 0.12 or less, respectively. These ranges have been proposed considering the criterium of von Mises ($IFSS=\sigma_{T}^{M}/\sqrt{3}$) and Tresca ($IFSS=\sigma_{T}^{M}/2$) criteria that correspond to the very well-bonded and well-bonded expected $f_{C}$ values, where IFSS is the interfacial shear strength.

### 3.3. The Analysis of the Chemical Composition and Microfibril Angle of Jute Strands

The content of lignin and hemicellulose, the main amorphous phases each one with a density of 1.40 g cm^−3^, decreased as the KN increased, as has been stated. Then, the amount of amorphous cellulose (density of 1.42 g cm^−3^) decreases, while the crystalline cellulose (density of 1.60 g cm^−3^) content increases after each delignification stage, as Table 4 shows. The densities of the lignin, hemicelluloses, and celluloses were recovered from [38]. However, other authors have indeed reported different densities for these components [39]. It was found that the CI of the jute strands increased with the subsequent delignification stages. Such behavior implies an increment of the crystalline regions of the fiber at the expense of the amorphous after each delignification stage. It is estimated that individual fibers composed of single cells with high cellulose content are obtained from the fiber bundles in higher amounts after each successive delignification stage [40]. The removal of lignin and hemicellulose was attributed to the presence of sodium hypochlorite during delignification [15]. Comparatively, the chemical composition of the raw jute strands is in line with that reported for different genotypes of *Corchorus capsularis* L. and *Corchorus olitorius* L. [41]. Jute is a strand (or phloem fiber) that contains a proportionally high amount of lignin in its fiber cell walls.

**Figure 5:**
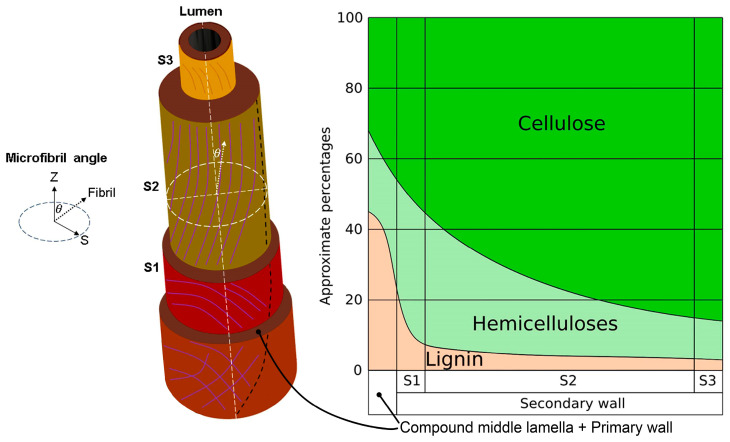
Schematic diagram of the three-dimensional structure and distribution of the main chemical components of a delignified fiber across the sections of the fiber cell wall, emphasizing the microfibril angle (θ) corresponding to the cellulose microfibrils located in the S2 layer.

The cellulose fibers provide mechanical support to the plant. As Figure 5 depicts, the plant fiber cells have a central lumen surrounded by the cell wall and are connected by the middle lamellae. In turn, the cell wall consists of a primary section followed by several secondary layers that form the thickest section (S1, S2, and S3) [42].

The cell wall layers contain lignin and hemicellulose regions intermixed with cellulose microfibrils disposed in a right-hand spiral. The angle between the fiber axis and the microfibrils in the S2 layer is denoted as the MFA, which is a key driver for the fiber and composite mechanical properties [39]. Thus, the cellulose microfibrils within the S2 are the main structural elements that govern the mechanical strength of the plant fibers, since it is estimated that the thickness of the S2 layer is more than a half of the cell wall [43]. The cell wall thickness scattering can be attributed to the different shapes and sizes of the fiber cells, as the reported micrographs reveal [41] If random cells are selected from the micrographs, a wide range of average wall thicknesses can be estimated for each cell, since their thickness varies considerably with the side measured (e.g., for three random cells and measuring the four sides of each one, the respective cell wall thickness was 16.67 ± 5.40 µm, 19.36 ± 4.22 µm, and 11.16 ± 3.26 µm). In response to this concern, another report estimated that the middle lamellae together with the lumen and the extreme cell wall layers (S1 and S3) of lignin-free cellulose fibers containing around 65 wt%, as is the case in the delignified jute strands, have relative thicknesses of 8%, while the S2 layer has 76% [43].

The individual cellulose fiber cells are composed of microfibrils whose orientation, specifically those from the S2 layer of the cell wall, have a significant influence on their fiber mechanical properties [44]. Indeed, cellulose microfibrils with small MFA facilitate the stress transfer from the matrix to the reinforcing fibers when the polymer composites are deformed [45]. Strands have the lowest MFA<10°, concerning fiber from leaves (10°–25°) and seeds (30–50°), leading to their corresponding high, moderate, and low level of mechanical strengths [42]. It has been reported that the MFA of lignocellulosic fibers does not significantly or consistently change after being treated with alkaline solutions or subjected to other treatments. Specifically, the MFA of cabuya (strands of leaves), fragrant screw pine, and ichu (grass) fibers respectively went from 6.6° to 7.1°, 7.2° to 8.1°, and 7.5° to 5.4° after being subjected to alkali treatment [44,45]). However, there is controversy on this matter since some authors have reported a change in the MFA or improvement of the intrinsic mechanical properties associated with the change of MFA during alkali treatment [46,47,48,49,50]. Thus, further investigation is needed.

For these cases, it is explained that progressive removal of hemicelluloses by the alkali treatment might involve a rearrangement of the network of cellulose microfibrils since these could be more susceptible to swelling and shrinkage that disoriented them, impacting the fiber-matrix stress transfer [14,51]. According to a reported graphical model, the cellulose microfibrils are interlinked by the electrostatic interactions between the functional groups of the pectin located between the S2 cellulose microfibrils and of the hemicellulose coating these microfibrils. It is speculated that the S2 layer hemicellulose content is negatively correlated to the MFA (higher hemicellulose content, lower MFA) since a more important hemicellulose matrix implies a larger interfibrillar space, which can absorb load and allows more sliding between cellulose microfibrils [52].

The sensitiveness of the fiber strength concerning the intrinsic physicochemical properties has been discussed in terms of the cellulose fraction, the volumetric cellulose crystallinity, the MFA, and the lumen porosity. According to a broad reported analysis of the sensitivity of the strength of jute strands, whose cell wall thickness goes from 8.304–11.341 µm [41] and cellulose microfibril width is 28 ± 3 Å as estimated from reported micrographs [53], and several other lignocellulosic fibers to MFA and other microstructural parameters, it has been found that, in general, the variations in MFA have little effect on the fiber strength variability. However, it is necessary to consider that the reported model predictions depend on the literature recovered MFA, which for jute strands varies from 7° to 9°, but the most representative is 8°, according to a broad literature review [39]. In turn, it has also been reported that the Cel(%) has a positive and strong correlation with the specific strengths, while negatively correlated with the MFA. On the contrary, the hemicellulose content has a negative correlation with the specific strengths and MFA. Meanwhile, the lignin content is directly proportional to the MFA, but inversely proportional to the specific strengths [54,55]. Thus, an increasing MFA decreases the strength and stiffness of the cell wall but increases the strain at the break. This phenomenon allows plants to adjust the mechanical behavior of their tissues by shifting the MFA [56].

### 3.4. The Analysis of the Impact of Chemical Composition on the Intrinsic Flexural Strength

In 1986, Mukherjee and Satyanarayana [51] established a series of linear and quadratic equations that relate the $\sigma_{f}^{F}$ to Cel(%). Since the literature shows a proportional relationship between $\sigma_{t}^{F}$ and $\sigma_{f}^{F}$, the authors explored further equations correlating the intrinsic flexural strength of jute strands ($\sigma_{f}^{F}$ and $\sigma_{f}^{F•}$) to MFA, Cel(%), and crystalline cellulose contents (CI⋅Cel(%)). The authors found that the highest coefficients of correlations were obtained for quadratic equations. The intrinsic flexural strength was positively correlated with the cellulose contents obtaining the following nonlinear regression quadratic Equations (6) and (7):

(6) $$\sigma_{f}^{F}=-1.8184{\left(Cel\%\right)}^{2}+292.94(Cel\%)-10540,\;\mathrm{with}\;0.91\;R^{2}$$

(7) $$\sigma_{f}^{F•}=-1.286{\left(Cel\%\right)}^{2}+209.3(Cel\%)-7319.6,\;\mathrm{with}\;0.95\;R^{2}$$

These equations show the positive correlation between cellulose content and the $\sigma_{f}^{F}$ of the strands. Moreover, the authors explored the correlation with the crystalline cellulose content (ψ=CI·Cel%), obtaining Equations (8) and (9):

(8) $$\sigma_{f}^{F}=-1.2752{\left(\psi \right)}^{2}+155.02(\psi )-3426.9,\;\mathrm{with}\;0.94\;R^{2}$$

(9) $$\sigma_{f}^{F•}=-0.9167{\left(\psi \right)}^{2}+112.89(\psi )-2260.6,\;\mathrm{with}\;0.98\;R^{2}$$

**Figure 6:**
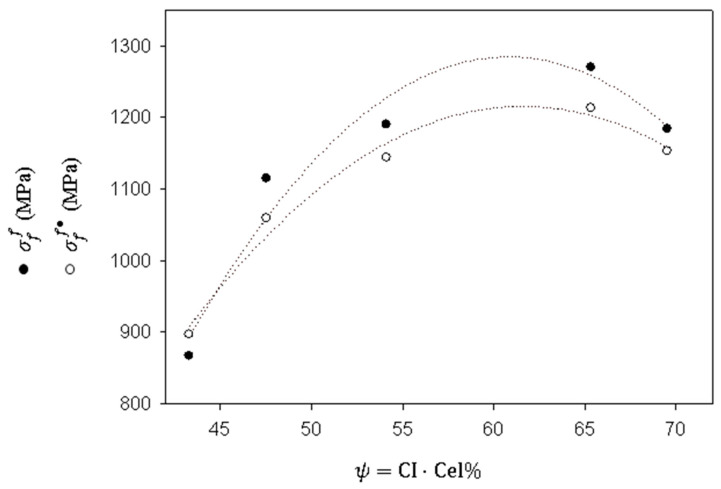
Intrinsic tensile strength of the jute strands as a function of crystalline cellulose contents and nonlinear regression quadratic curves obtained from the data (shown as dotted regression lines).

Thus, the intrinsic flexural strength of jute strands was more strongly correlated to crystalline cellulose contents than to whole cellulose content. Figure 6 shows the regression curves of Equations (8) and (9).

Considering that the literature stated the impact of MFA on the $\sigma_{t}^{F}$ of strands, the authors explored possible correlations to MFA. Mukherjee and Satyanarayana (1986) [51] studied the correlation of cos(MFA) to the intrinsic tensile strength of the strands. Thus, the authors explored the correlation of δ=Cel%·CI·cos(MFA) to the intrinsic flexural strengths, obtaining Equations (10) and (11):

(10) $$\sigma_{f}^{F}=-1.3004{\left(\delta \right)}^{2}+156.55(\delta )-3426.9,\;\mathrm{with}\;0.94\;R^{2}$$

(11) $$\sigma_{f}^{F•}=-0.9348{\left(\delta \right)}^{2}+114(\delta )-2260.6,\;\mathrm{with}\;0.98\;R^{2}$$

The inclusion of cos(MFA) in the equations did not alter the coefficients of determination. The authors used an MFA=8°, based on literature [45,48]. The low value of such an angle compared to the other factors and the fact that such cos(MFA) was constant are the main reasons for obtaining the same coefficients of correlation. The $\sigma_{f}^{F}$ is inversely correlated to MFA, thus, the authors explored the correlation of $\sigma_{f}^{F}$ to γ=CI·Cel%/MFA, obtaining Equations (12) and (13):

(12) $$\sigma_{f}^{F}=-81.612{\left(\gamma \right)}^{2}+1240.2(\gamma )-3426.9,\;\mathrm{with}\;0.94\;R^{2}$$

(13) $$\sigma_{f}^{F•}=-58.666{\left(\gamma \right)}^{2}+903.16(\gamma )-2260.6,\;\mathrm{with}\;0.98\;R^{2}$$

The results were like those obtained for cos(MFA), and for the same reasons. To evaluate the possible impact of MFA variations on the $\sigma_{f}^{F}$ of the jute strands, and taking into account, on the one hand, that MFA for jute varies from 7° to 12° [57], which is in line with the separation of nanofibrils across the cell wall [58], and on the other hand that MFA can increase due to the harshness of the treatments, the authors applied a range of MFA from 8° to 12° to J.0 to J.4, respectively (see Table 4). Using the equation that correlated the intrinsic flexural strengths with δ, the coefficients of correlations decreased to 0.91 and 0.95. These equations revealed that the intrinsic strength of the reinforcements is positively correlated with the crystalline cellulose contents of the strands and inversely correlated to the MFA. These equations can be used to explore the impact of these parameters on the intrinsic flexural strength, but not for evaluating the properties.

### 3.5. The Analysis of the Specific Flexural Properties of the Polylactic Acid, Its Composites, and Reinforcing Jute Strands

The potential selection of PLA composites for light-mass applications, such as automotive, requires not only the corroboration of flexural properties suitable for products and parts subjected to bending loads but also requires the consideration of specific mechanical properties (e.g., the ratio between flexural strength and the material density). Dividing the mechanical properties of the material by its density normalizes them, allowing the objective cost comparison with other materials [59]. Specifically, the comparison of specific mechanical properties can be used to analyze weight savings, which is important in the automotive and aerospace industries [60]. As expected, the addition of jute strands to PLA led to specific $\sigma_{f}^{C}$ values higher than the PLA matrix by 15%, 39%, 38%, 44%, and 38%, respectively, for the composites reinforced with raw jute and subsequently delignified strands (see Table 5). These enhancement percentages are comparable with others reported for hemp fiber-reinforced PLA composites [61]. Moreover, the jute strands-reinforced composites respectively achieved 64%, 78%, 77%, 80%, and 77% of the specific $\sigma_{f}^{C}$ of the commercially available GF-reinforced PLA composite. The remarkable specific flexural performance of the PLA composites is proportional to the specific $\sigma_{f}^{F}$ of the reinforcing jute strands.

Then, the potential practical implication of these findings is a positive effect on the future development trends in PLA biocomposites. For instance, the improvement of the PLA composites manufacturing based on different types, ratios, and shapes of natural fibers for specific applications. These results could contribute to the development of proper databases on natural fibers and biocomposites [1].

## 4. Conclusions

Herein, cellulose fiber-reinforced biocomposites have been manufactured and characterized. The delignification stages using sodium hypochlorite generate, apart from the decrease in lignin content, the progressive increase of cellulose in the delignified strands. The hemicelluloses and amorphous material content decreases, also progressively, given that the delignifying agent is not selective for lignin, unlike sodium chlorite. It is also evident that most of the extractives present in the primary wall, mainly, disappear, which notably favors the IFSS of the composite materials. Because of the change in the chemical composition of the strands, the $\rho^{F}$ increases slightly and progressively up to 4% concerning the raw jute strands.

The $\sigma_{f}^{C}$ increases significantly after the first delignification stage concerning the PLA matrix and composite manufactured with raw jute strands, reaching a stable behavior or “plateau” of around 100 MPa from the second delignification stage. The composite manufactured with strands subjected to the third delignification stage reaches its maximum value of 104.55 MPa. This value represents 66% and 61.3% of the $\sigma_{f}^{C}$ of two PLA composites reinforced with GF. The evolution of the $\sigma_{f}^{C}$ correlates reasonably well with the $\sigma_{t}^{C}$.

The intrinsic flexural strengths obtained from the relation between FTSM and FFSM and from the ratio between the flexural and tensile strengths of the composites show a good correlation. The intrinsic flexural strengths show a fair correlation to the intrinsic tensile strengths.

Concerning the intrinsic mechanical properties, nonlinear equations that correlate the intrinsic flexural strength of the jute strands ($\sigma_{f}^{F}$ and $\sigma_{f}^{F•}$) with the CI, Cel%, and MFA were obtained for a constant MFA=8° with respect the delignification stages. To the best knowledge of the author, these correlations are a novelty for the prediction of the intrinsic mechanical strength of jute strands.

Finally, the specific $\sigma_{f}^{C}$ of the jute strands-reinforced PLA composites achieved more than 70% of commercially available PLA reinforced with GF. In addition, the lower and lesser scattered specific price of jute strands, and their environmental and processing advantages, indicate that these PLA composites could be considered for manufacturing products that need to be subjected to bending forces such as some GF-reinforced PLA products.

## Figures and Tables

**Table 1:** Structural properties and chemical composition of the raw and delignified jute strands.

Stages	$\rho^{F}$ (g cm^−3^)	D (μm) ^1^	KN ^1^	Lignin (%)	Hemicellulose (%)	Cellulose (%)	CI (%)
J.0	1.48	22.90	27.3 ± 2.5	13.0	18.3	66.6	65.0
J.1	1.50	22.80	20.0 ± 1.7	9.2	16.1	69.8	68.1
J.2	1.51	22.60	14.8 ± 1.5	7.5	15.8	77.3	70.0
J.3	1.53	22.55	7.7 ± 0.8	3.9	13.6	82.7	79.0
J.4	1.54	22.40	2.1 ± 0.6	0.8	13.1	86.9	80.0

^1^ Recovered from [15].

**Table 2:** Flexural mechanical properties of the PLA composites reinforced with jute strands (J.0) and delignified strands (J.1–J.4) and comparison with their tensile mechanical properties and of reported GF-reinforced PLA composites.

Stages	wt%	$V^{F}$	$\sigma_{f}^{M}\vee \sigma_{f}^{C}$ (MPa) ^4^	$\sigma_{t}^{M}\vee \sigma_{t}^{C}$ (MPa) ^1,4^	$\varepsilon_{f}^{M}\vee \varepsilon_{f}^{C}$ (MPa) ^4^	$\sigma_{f}^{M*}$ (MPa)
NA	0	0	68.35 ± 0.90 ^a^	49.8 ± 1.54 ^a^	3.27 ± 0.40 ^a^	NA
J.0	30	0.264	82.15 ± 3.20 ^b^	54.7 ± 2.75 ^b^	2.42 ± 0.20 ^b^	61.52
J.1	30	0.262	100.05 ± 2.40 ^c^	68.6 ± 0.54 ^c^	2.62 ± 0.04 ^b^	65.46
J.2	30	0.260	99.35 ± 2.90 ^c^	70.0 ± 0.84 ^d^	2.48 ± 0.04 ^b^	62.75
J.3	30	0.258	104.55 ± 1.70 ^d^	72.9 ± 1.08 ^e^	2.54 ± 0.04 ^b^	63.94
J.4	30	0.257	100.55 ± 2.70 ^c^	71.7 ± 1.53 ^e^	2.55 ± 0.05 ^b^	64.13
GF ^2^	30	0.173	140	92	0.90	NA
GF ^3^	30	0.173	145	114	NA	NA

^1^ Recovered from [15]. ^2^ and ^3^ are values, respectively, obtained from the data reported by [26] ($\sigma_{f}^{M}=82\;MPa$ and $\sigma_{t}^{M}=43\;MPa$) and [27] ($\sigma_{f}^{M}=108\;MPa$ and $\sigma_{t}^{M}=62\;MPa$). GF exhibited ($\rho^{F}=2.55\;g\;{cm}^{-3}$). ^4^ Different letters a, b, c, d, and e represent the statistical difference (ANOVA, *p* < 0.05) between the properties of the materials.

**Table 3:** Values of $\sigma_{f}^{F}$ of the jute strands reinforcing the PLA estimated through $\sigma_{f}^{F}=\sigma_{t}^{F}\frac{FFSF}{FTSF}$ or $\sigma_{f}^{F•}=\sigma_{t}^{F}\frac{\sigma_{f}^{C}}{\sigma_{t}^{C}}$. The $f_{C}$ and $f_{C}^{•}$ were estimated from $\sigma_{f}^{F}$ and $\sigma_{f}^{F•\prime }$, respectively.

Stages	wt%	$V^{F}$	FFSF (MPa)	FTSF (MPa) ^1^	$\sigma_{t}^{F}$ (MPa) ^1^	$\sigma_{f}^{F}$ (MPa)	$f_{C}$	$\sigma_{f}^{F•}$ (MPa)	$f_{C}^{•}$
J.0	30	0.264	139.66	96.20	597	867	0.161	897	0.156
J.1	30	0.262	197.48	128.60	726	1115	0.177	1059	0.187
J.2	30	0.260	203.52	137.80	806	1190	0.171	1144	0.178
J.3	30	0.258	221.34	147.50	846	1270	0.174	1213	0.182
J.4	30	0.257	205.84	142.90	822	1184	0.174	1153	0.179
GF ^2^	30	0.173	417.26	326.24	2400	3069	0.136	3652	0.114
GF ^3^	30	0.173	321.87	362.58	2400	2131	0.151	3053	0.105

^1^ Recovered from [15]. ^2^ and ^3^ are values, respectively, obtained from the data reported by [26] and [27]. GF exhibited. The $\sigma_{t}^{F}$ value of GF corresponds to sized fibers.

**Table 4:** Crystalline and amorphous cellulose content and MFA of the delignified jute strands.

Stages	CI (%)	Cellulose (%)			MFA (°)	
		Total	Crystalline	Amorphous	Constant	Variable
J.0	65.0	66.6	43.3	23.3	8	8
J.1	68.1	69.8	47.5	22.3	8	9
J.2	70.0	77.3	54.4	22.9	8	10
J.3	79.0	82.7	65.3	17.4	8	11
J.4	80.0	86.9	69.5	17.4	8	12

**Table 5:** Specific flexural mechanical properties of the PLA, its composites, and the delignified jute strands.

Stages	wt%	$V^{F}$	$\rho^{M}\vee \rho^{C}$ (g cm^−3^)	$\frac{\sigma_{f}^{M}}{\rho^{M}}\vee \frac{\sigma_{f}^{C}}{\rho^{C}}$ (MPa)	$\frac{\varepsilon_{f}^{M}}{\rho^{M}}\vee \frac{\varepsilon_{f}^{C}}{\rho^{C}}$ (%)	$\rho^{F}$ (g cm^−3^)	$\frac{\sigma_{f}^{F}}{\rho^{F}}$ (MPa)
NA	0	0	1.24	55.1	2.64	NA	NA
J.0	30	0.264	1.30	63.2	1.85	1.48	585.6
J.1	30	0.262	1.31	76.4	2.00	1.50	743.3
J.2	30	0.260	1.31	75.8	1.88	1.51	788.3
J.3	30	0.258	1.32	79.2	1.93	1.53	829.8
J.4	30	0.257	1.32	76.2	1.93	1.54	768.9
GF ^1^	30	0.173	1.47	98.6	NA	2.55	941.2

^1^ Recovered from [27].

## Data Availability

Data are contained within the article.

## Abbreviations

ψ	The product of the crystallinity index and the cellulose content
δ	The product of the crystallinity index, the cellulose content, and the microfibrillar angle cosine
γ	The product of the crystallinity index and the cellulose content, divided by the microfibrillar angle.
l⁄d	length-diameter ratio
V	Volume of the pycnometer
D	Fiber diameter
S3	Inner secondary layer of the cell wall
S2	Middle secondary layer of the cell wall
S1	Outermost secondary layer of the cell wall
PLA	Poly-(lactic acid)
MFI	Melt flow index
MFA	Microfibrillar angle
KN	Kappa number
J.4	Jute strands after four subsequent delignification stages
J.3	Jute strands after three subsequent delignification stages
J.2	Jute strands after two subsequent delignification stages
J.1	Jute strands after one delignification stage
J.0	Raw jute strands
IFSS	Interfacial shear strength
GF	Glass fibers
FTSF	Fiber tensile strength factor
FFSF	Fiber flexural strength factor
CI	Crystallinity index
Cel%	Cellulose content
$\sigma_{t}^{F}$	Fiber tensile strength
$\sigma_{t}^{C}$	Composite tensile strength
$\sigma_{f}^{m*}$	Contribution of the matrix to the composite flexural strength
$\sigma_{f}^{M}$	Matrix flexural strength
$\sigma_{f}^{F•}$	Intrinsic flexural strength obtained from the ratio of flexural and tensile strengths.
$\sigma_{f}^{F}$	Intrinsic flexural strength
$\sigma_{f}^{C}$	Composite flexural strength
$\rho^{F}$	Fiber density
$\rho^{C}$	Composite density
$\rho^{M}$	Matrix density
$\rho^{H_{2}O}$	Distilled water density
$\varepsilon_{f}^{M}$	Elongation at break of the matrix under flexural load
$\varepsilon_{f}^{C}$	Elongation at break of the composite under flexural load
$w^{M}$	Matrix weight
$w^{F}$	Fiber weight
$w^{C}$	Composite weight
$w^{H_{2}O}$	Distilled water weight
$f_{C}^{•}$	Flexural coupling factor obtained from $\sigma_{f}^{F•}$
$f_{C}$	Flexural coupling factor
$V^{F}$	Fiber volume fraction
